# The regulatory role and mechanism of lncTUG1 on cartilage apoptosis and inflammation in osteoarthritis

**DOI:** 10.1186/s13075-023-03087-7

**Published:** 2023-06-20

**Authors:** Nan-nan Liu, Yan-ping Huang, Yu-bao Shao, Xue-fei Fan, He-yan Sun, Tao-rong Wang, Tao Yao, Xiao-Yu Chen

**Affiliations:** 1 Department of Histology and Embryology, Anhui Medical University, No. 81 Meishan Road, Hefei, 230032 Anhui Province China; 2 Department of Human Anatomy, Histology and Embryology, Anhui Medical College, No. 632 Furong Road, Hefei, 230601 Anhui Province China; 3 Department of Orthopedics, The First Affiliated Hospital of Anhui Medical University, No. 218 Jixi Road, Hefei, 230022 Anhui Province China; 4 Department of Orthopedics, The Third Affiliated Hospital of Anhui Medical University, No. 390 Huaihe Road, Hefei, 230061 Anhui Province China

**Keywords:** Osteoarthritis, TUG1, miR-144-3p, DUSP1, P38 MAPK

## Abstract

**Background:**

Long-stranded non-coding RNA TUG1 is lowly expressed in osteoarthritic chondrocytes. This study aimed to elucidate the role of TUG1 in osteoarthritic cartilage damage and the underlying mechanisms.

**Methods:**

Combined database analysis, using primary chondrocytes as well as the C28/I2 cell line, was performed by qRT-PCR, Western blotting, and immunofluorescence to determine the expression of TUG1, miR-144-3p, DUSP1, and other target proteins. Dual luciferase reporter gene and RIP to verify direct interaction of TUG1 with miR-144–3-p and miR-144–3-p with DUSP1, Annexin V-FITC/PI double staining to detect apoptosis. CCK-8 to detect cell proliferation. The biological significance of TUG1, miR-144-3p, and DUSP1 was assessed in vitro experiments using siRNA for TUG1, mimic and repressor for miR-144-3p, and overexpression plasmid for DUSP1. In this study, all data were subjected to a *t*-test or one-way analysis of variance with a *p*-value < 0.05 as the cutoff.

**Results:**

TUG1 expression was closely associated with osteoarthritic chondrocyte damage, and knockdown of TUG1 significantly promoted chondrocyte apoptosis and inflammation. In the present study, we found that TUG1 inhibited chondrocyte apoptosis and inflammation by competitively binding miR-144-3p, deregulating the negative regulatory effect of miR-144-3p on DUSP1, promoting DUSP1 expression, and inhibiting the p38 MAPK signaling pathway.

**Conclusions:**

In conclusion, our study clarifies the role of the ceRNA regulatory network of TUG1/miR-144-3p/DUSP1/P38 MAPK in OA cartilage injury and provides an experimental and theoretical basis for genetic engineering tools to promote articular cartilage repair.

**Graphical abstract:**

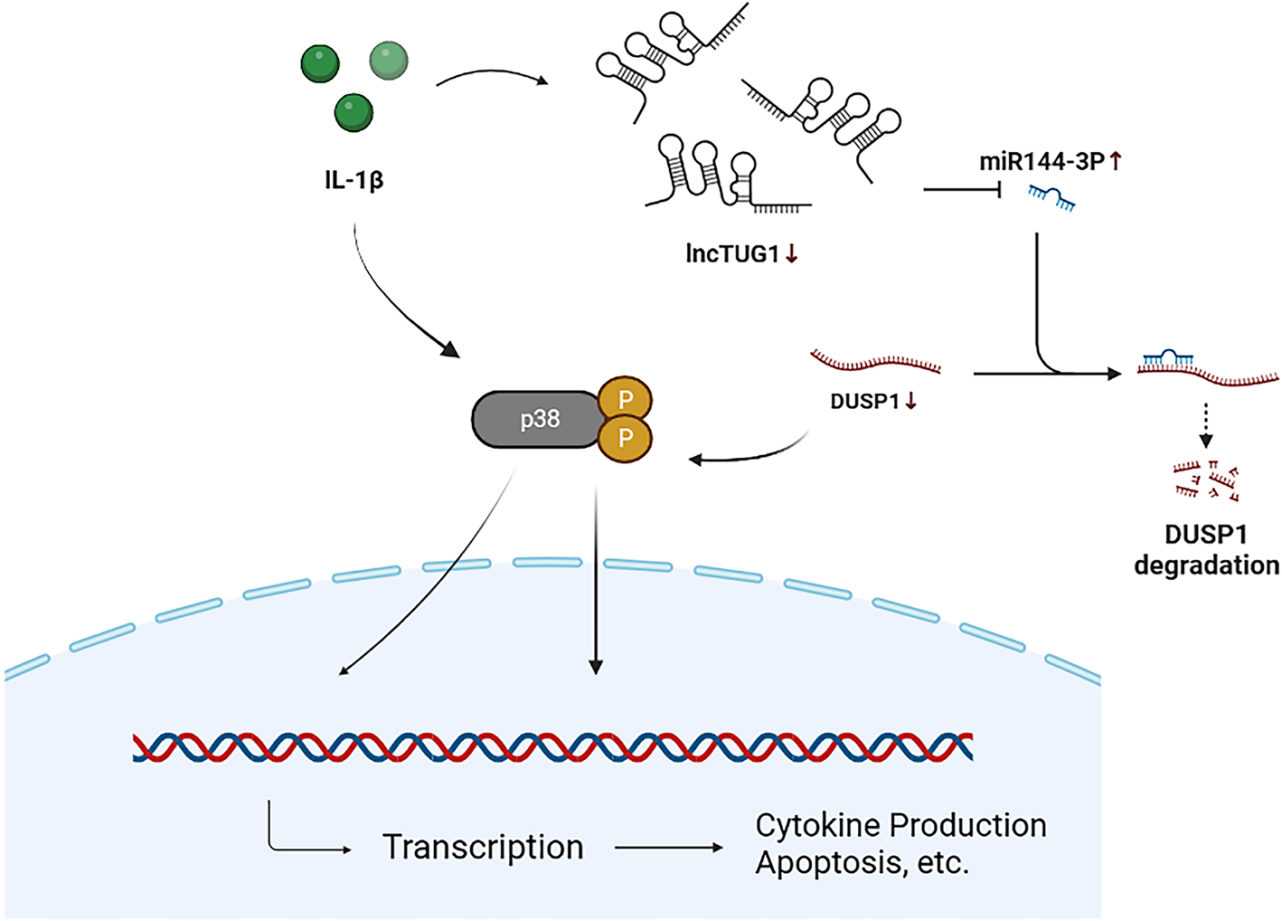

**Supplementary Information:**

The online version contains supplementary material available at 10.1186/s13075-023-03087-7.

## Introduction

Osteoarthritis (OA) is one of the most common joint diseases, especially in people over 60 years of age [1]. Chondrocytes are the only cells in cartilage tissue whose physiological functions such as apoptosis, inflammation, and autophagy play an important regulatory role in the process of cartilage degeneration [2]. Recent studies have revealed that lncRNAs play an important regulatory role in the pathological process of OA. According to Bai et al. [3], lncHCAR accelerates osteoarthritic cartilage degeneration by competitively binding miR-15b-5p and increasing the expression of Vegfa and Mmp13. Additionally, in vitro chondrocyte models of OA produced by inflammatory factors have revealed many lncRNAs linked to cartilage apoptosis and inflammation, including SNHG7 [4–6]. In the chromosome 22q12 area, lncRNA TUG1 (taurine upregulated 1) was first identified as being important in the formation of photoreceptors and the retina. Subsequent research showed that TUG1 also regulates cell proliferation, differentiation, and apoptosis [7–9]. TUG1 was recently discovered to be expressed abnormally in some tumors, impacting the proliferation and apoptosis of tumor cells [10–12]. However, TUG1 was shown to be low expressed in IL-1-induced OA chondrocytes in our study, indicating that TUG1 downregulation may promote OA chondrocyte damage.

The competitive endogenous RNAs (ceRNAs) can bind to miRNAs and affect miRNA-induced gene silencing [13]. Numerous studies have demonstrated that lncRNAs can function as ceRNAs to bind miRNAs and take part in the control of gene expression [14]. Using databases like StarBase 2.0, it was predicted that TUG1 and miR-144-3p had binding sites, and miR-144-3p appeared to be highly expressed after interference with TUG1, indicating that it may be a direct target molecule of TUG1. In chondrocytes, H19 can act as a ceRNA to competitively bind miR-675 to promote type II collagen expression and maintain chondrocyte stability [15]. Additionally, it has been shown that OA chondrocytes produce a high level of miR-144-3p [16], which raises the possibility that TUG1 can reduce OA chondrocyte apoptosis and inflammatory response by directly binding to miR-144-3p and suppressing miR-144-3p expression.

Dual-specificity phosphatase 1 (DUSP1) is an important negative regulator of the inflammatory response. It was shown that DUSP1 can inhibit osteoarthritis by suppressing the activation of the p38 MAPK signaling pathway and exerting anti-inflammatory and anti-catabolic actions [17]. According to databases like StarBase 2.0, DUSP1 is weakly expressed in IL-1-induced chondrocytes and its 3′UTR can bind to miR-144-3p, predicting that miR-144-3p may regulate the p38 MAPK signaling pathway by inhibiting DUSP1, thereby regulating chondrocyte apoptosis and inflammation.

Studies have confirmed that inhibition of p38 MAPK expression or blockade of the p38 MAPK signaling pathway during inflammation can significantly slow down cartilage degeneration, inhibit inflammatory response, and improve OA cartilage damage [18, 19]. Our study also confirmed that p38 MAPK was activated in IL-1β-treated chondrocytes, and analysis of the correlation between TUG1 and p38 MAPK expression revealed a significant negative correlation, suggesting that TUG1 may inhibit chondrocyte apoptosis and inflammatory response by suppressing the p38 MAPK signaling pathway. In summary, we propose that TUG1 may enhance the expression of DUSP1 and inhibit the p38 MAPK signaling pathway by competitively binding to miR-144-3p and releasing DUSP1 from miR-144-3p’s negative regulation, thereby inhibiting OA chondrocyte apoptosis and inflammatory response and reducing cartilage degeneration and damage.

## Materials and methods

### Clinical samples

Eleven specimens of knee cartilage were collected from the First Affiliated Hospital of Anhui Medical University in patients with OA who underwent knee replacement surgery, and all patients were diagnosed with OA according to the OA diagnostic guidelines. All clinical data were obtained with the approval and consent of the clinical management committee of our hospital. According to Article 9 of the ethical review application/reporting guidelines of the Clinical Medical Research Ethics Committee of Anhui Medical University, the study posed no greater than minimal risk to the subjects, protected the privacy of the subjects, followed the subjects’ wishes to be exempted from signing written informed consent documents, and it has been reviewed by the Biomedical Ethics Committee of Anhui Medical University (S20200024).

### Cell lines and cell culture

Primary chondrocytes and C28/I2 cell lines (from the First Affiliated Hospital of Anhui Medical University) were cultured in high-glucose DMEM (HDMEM, WISENT, Canada) with 10% fetal bovine serum (WISENT, Canada) and 1% antibiotics (penicillin–streptomycin, Beyotime Biotechnology, Shanghai, China). Human OA articular chondrocytes were cultured in HDMEM and used for the study after 1–3 passages. All cells were cultured at 37 °C and 5% CO_2_. To treat the cells, recombinant human IL-1β was obtained from PeproTech (NJ, USA).

### Cell transfection and processing

After the cell fusion rate reached 60%, Si-TUG1, miR-144-3p mimic, miR-144-3p inhibitor, and DUSP1 overexpression plasmids were transfected with Lipofectamine 2000 (Thermo Fisher Scientific, MA, USA) into After 6 h, the medium was replaced with fresh medium, and the culture was continued for 24 h. After transfection, cells were induced with 10 ng/ml IL-1β for 24 h and used for subsequent assays. Si-TUG1 was purchased from Hanbio Biotechnology (Shanghai, China), miR-144-3p mimic, miR-144-3p inhibitor, and DUSP1 overexpression plasmids were purchased from Tsingke Biotechnology (Beijing, China).

The sequences of Si-TUG1, mimics, and inhibitors are shown in Table 1.

**Table 1:** The sequences of Si-TUG1, mimics, and inhibitors

Name	Sequences
Si1-TUG1	Sense: GGCCUAUGCGUUUGCGAUUTT
	Antisense: AAUCGCAAACGCAUAGGCCTT
Si2-TUG1	Sense: GGAUGGUUGGUUGUGGGAUTT
	Antisense: AUCCCACAACCAACCAUCCTT
Si3-TUG1	Sense: GUUGGUUGUGGGAUUUCUATT
	Antisense: UAGAAAUCCCACAACCAACTT
Si-NC	Sense: UUCUCCGAACGUGUCACGUTT
	Antisense: ACGUGACACGUUCGGAGAATT
miR-144-3p-mimic	Sense: UACAGUAUAGAUGAUGUACU
	Antisense: UACAUCAUCUAUACUGUAUU
miR-144-3p-inhibitor	Sense: AGUACAUCAUCUAUACUGUA

### Dual-luciferase reporter assay

Based on the predicted results, 2 luciferase reporter vectors (Genechem, Shanghai, China) were constructed: PGL3-TUG1 RNA (containing a binding site to miR-144-3p) and PGL3-TUG1 RNA mut (containing a mutation site binding to miR-144-3p). Cell spreading was performed 24 h before transfection, with 1 × 10^4^ cells per well in 96-well culture plates. Then, miR-144-3p mimic or inhibitor was cotransfected with the above two luciferase reporter vectors, respectively. Forty-eight hours after transfection, cells were rinsed twice with PBS, 100 μl of lysis solution was added to each experimental well for 20 min, the double luciferase reporter assay kit (TransGen Biotech, Beijing, China) was performed to determine the dual luciferase activity, and Firefly luciferase/Rennia luciferase (enzyme activity) was used to indicate the relative transcriptional activity of reporter genes.

### RNA immunoprecipitation (RIP)

RIP was used to verify the binding relationship between TUG1 and miR-144-3p or miR144-3P and DUSP1. Whole-cell lysates were incubated overnight at 4 °C with RIP buffer containing magnetic beads (GenScrip, NJ, USA) coupled with anti-Ago2 antibody (Proteintech, Wuhan, China), and immunoglobulin G (IgG) was used as a negative control. The beads were washed, and RNA was extracted. Co-precipitated RNA was evaluated by qRT-PCR.

### Cell counting kit-8 (CCK-8) assay

Chondrocytes at the logarithmic growth stage were harvested and inoculated in 96-well plates at a density of 1 × 10^3^/ml per well, and the edge wells were filled with PBS and incubated with 5% CO_2_ at 37 °C until the cells were adherent. After 24 h of transfection, 10 μl of CCK-8 solution (Biogene Medical Technology, Anhui, China) was added to each well. The plates were incubated in the incubator for 1–4 h. The absorbance at 450 nm was measured with an enzyme marker.

### Flow cytometric analysis

Cells were inoculated into 6-well plates at a density of 5 × 10^5^/ml for 24 h. Si-TUG1 was transfected and then treated with 10 ng/ml IL-1β for 24 h. Cells were digested by adding 0.25% trypsin solution (without EDTA), washed with PBS, centrifuged, and resuspended by adding 100 μl of the buffer. Then, 5 μl Annexin V-FITC and 5 μl PI (Bestbio, Shanghai, China) were added and incubated for 15 min at room temperature and protected from light; 400 μl of buffer was added, and the cells were measured by flow cytometry (BD Celesta, USA).

### Hochest33342 staining

Discard the medium, wash twice with PBS, add dropwise an appropriate amount of Hoechst 33,342 Live Cell Staining Solution (Beyotime Biotechnology, Shanghai, China), and mix gently. Incubate for 10 min at a temperature suitable for cell culture. Aspirate the staining solution, wash 2–3 times with culture medium or PBS, and observe under the fluorescence microscope.

### Western blotting analysis

Cells were collected, total cellular proteins were extracted using RIPA (Beyotime Biotechnology, Shanghai, China), and cell lysates were collected in EP tubes, sonicated three times, and centrifuged for 5 min in an ice bath at 12,000 rpm, 4 °C. The supernatant was transferred to another EP tube and BCA (Thermo Fisher Scientific, MA, USA) for protein quantification. The extracted proteins were added to the loading buffer and cooked at 95 °C for 5 min. Ten percent polyacrylamide gel electrophoresis was performed to separate the proteins with a loading volume of 30 μg per well. Electrophoresis was performed at 80 to 120 V, wet rotation, and 200 mA rotational membrane current for 120 min. Five percent skim milk powder PBS was closed at room temperature for 2 h. The indicated primary antibodies were added and incubated overnight at 4 °C. Primary antibodies were diluted in PBST as follows: β-actin (primary antibody: PBST = 1:1000, Boster, Wuhan, China), collagen II, MMP13, Bax, p-p38 (primary antibody: PBST = 1:1000, Immunoway, TX, USA), Dusp1 (primary antibody: PBST = 1:1000, Bioss, Beijing, China), Bcl2, p38 (primary antibody: PBST = 1:1000, Abmart, Shanghai, China), caspase3 (PBST was rinsed 3 times/10 min, incubated with the corresponding secondary antibody for 1 h at room temperature, washed 4 times/10 min, and developed by chemiluminescence reagent (New Cell & Molecular Biotech, Suzhou, China) Shanghai, China). The target bands were analyzed by the ImageJ software for grayscale values.

### Immunofluorescence

Cells were fixed in 4% paraformaldehyde, cleared with 0.3% Triton-X 100-PBS, and closed with 5% BSA for 1 h. Cells were incubated with the indicated primary antibody as well as the corresponding fluorescently labeled secondary antibody (Immunoway, TX, USA). Fluorescence imaging was performed with an ortho-fluorescence microscope (Leica, Germany).

### Total RNA isolation and quantitative real-time reverse transcription PCR (qRT-PCR)

RNA was extracted from chondrocytes using an RNA extraction kit (Accurate Biology, Hunan, China), and the RNA concentration was detected by UV spectrophotometer and stored at − 80 ℃. The expression of TUG1, DUSP1, miR-144-3p, MMp13, and collagen II was detected by SYBR Green QPCR master mix (Beyotime Biotechnology, Shanghai, China). Using β-actin as the internal reference control, the average CT value (amplification power curve inflection point) was taken and semi-quantitative analysis was performed by 2^−△△Ct^ to calculate the relative expression of target genes. The primer sequences are shown in Table 2.

**Table 2:** The primer sequences

Gene	Primer sequence
Collagen II	Forward: GAGCCCTGCCGGATCTGT Reverse: GAGGCAGTCTTTCACGTCTTC
MMP13	Forward: TCCTGATGTGGGTGAATACAATG Reverse: GCCATCGTGAAGTCTGGTAAAAT
TUG1	Forward: CACTCATCCTGTGCCTCCTG Reverse: TGATGGCTGAATGCCTCCTG
miR144-3P	RT GTCGTATCCAGTGCAGGGTCCGAGG TATTCGCACTGGATACGACAGTACA Forward: GCGCGCTACAGTATAGATGA Reverse: GTGCAGGGTCCGAGGT
Dusp1	Forward: GCATGGTCATGGAAGTGGGCAC Reverse: GTCAGCAGCTGGGAGAGGTCG
β-Actin	Forward: CTCCATCCTGGCCTCGCTGT Reverse: GCTGTCACCTTCACCGTTCC
U6	RT AACGCTTCACGAATTTGCGT Forward: CTCGCTTCGGCAGCACA Reverse: AACGCTTCACGAATTTGCGT

### Statistical analysis

In the present study, three samples were required for all tests. Prism 8.0 (GraphPad, San Diego, CA) was applied for data analysis, with a *t*-test or one-way analysis of variance, using a *p*-value < 0.05 as a cutoff. Continuous variables are shown as mean ± standard error (SEM).

## Results

### Low expression of TUG1 in human osteoarthritis primary chondrocytes

**Fig. 1:**
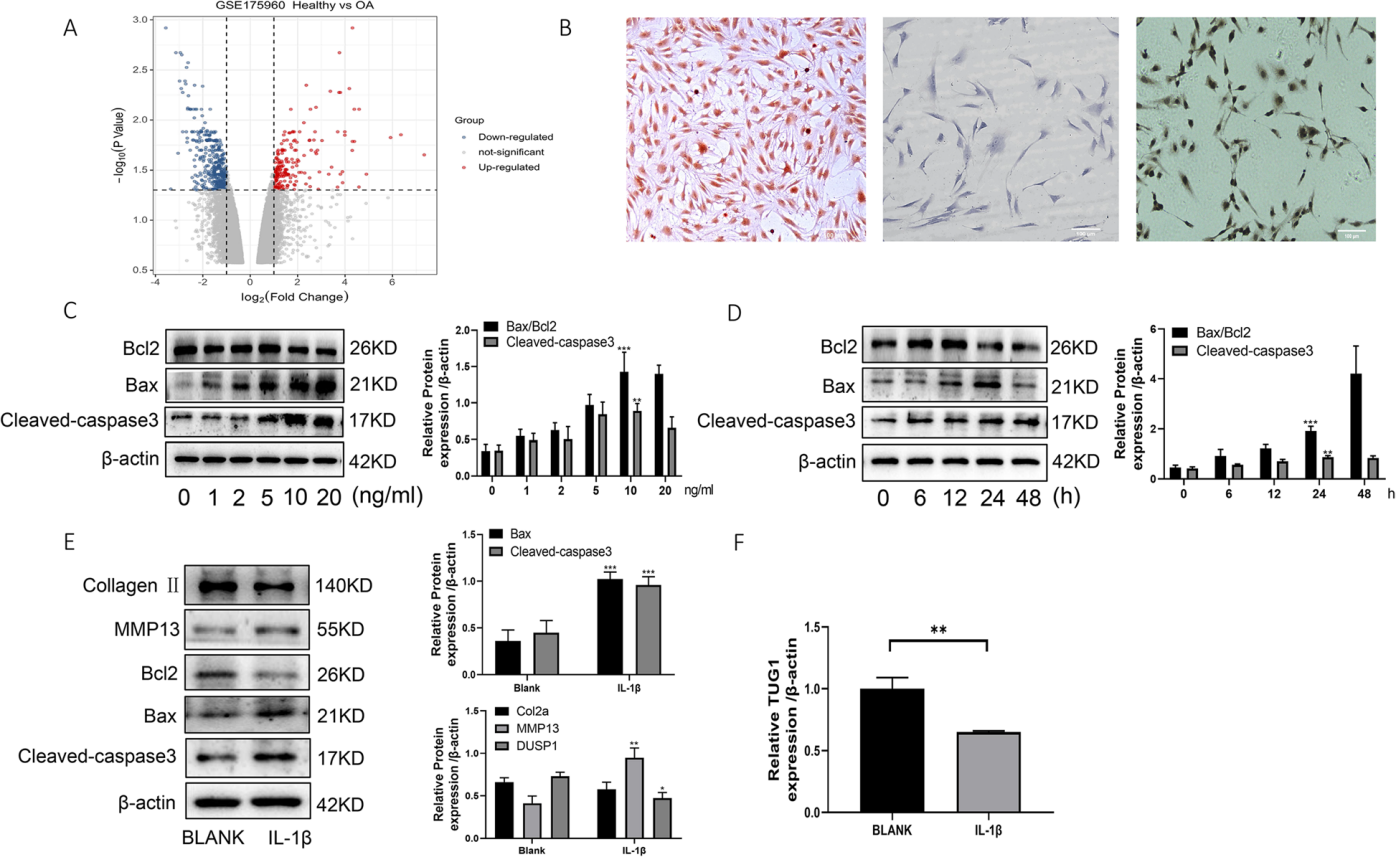
TUG1 is lowly expressed in human osteoarthritic primary chondrocytes. **A** Differential analysis of the GEO database dataset (GSE175960) using the limma package. **B** Identification of primary chondrocytes by Saffron-O and Fast Green, toluidine blue, and collagen II immunocytochemistry staining. **C** Western blotting to detect the protein expression of Bax, Bcl2, and cleaved-caspase3 under different concentrations of IL-1β stimulation. **D** Western blotting to detect the protein expression of Bax, Bcl2, and cleaved-caspase3 after different times of IL-1β stimulation. **E** Western blotting to detect the protein expression of collagen II, MMP13, Bax, Bcl2, and cleaved-caspase3 after 24-h stimulation with 10 ng/ml IL-1β. **F** qRT-PCR assay of TUG1 mRNA expression after 24-h stimulation of chondrocytes using 10 ng/ml IL-1β. All data are shown as SEM ± mean based on 3 independent experiments. *P < 0.05; **P < 0.01; *P* < 0.001

To investigate the role of TUG1 in OA, differential analysis based on published microarray data GSE175960 (from the GEO database) yielded 253 upregulated genes and 475 downregulated genes, with TUG1 showing significant downregulation (Fig. 1A). To further investigate the changes in TUG1 in OA, OA patients with total knee replacement were selected, and chondrocytes were extracted from the knee cartilage and identified using Saffron-O and Fast Green Stain, toluidine blue, and collagen II immunocytochemistry staining (Fig. 1B). The chondrocytes were treated with different concentrations (0, 1, 2, 5, 10, 20 ng/ml) of IL-1β for 24 h, followed by treatment of chondrocytes with 10 ng/ml IL-1β for different times (0, 6, 12, 24, 48 h), and protein expression associated with apoptosis was examined (Fig. 1C, D). The optimal treatment concentration and time (10 ng/ml, 24 h) were selected to treat OA chondrocytes, and the expression of apoptosis and cartilage damage-related proteins were detected (Fig. 1E). qRT-PCR was performed to detect the expression of TUG1 in OA chondrocytes treated with IL-1β, and the expression of TUG1 was found to be downregulated (Fig. 1F). The results suggest that TUG1 may be involved in chondrocyte apoptosis and inflammation.

### TUG1 is downregulated in IL-1β-induced C28/I2 chondrocyte lines

**Fig. 2:**
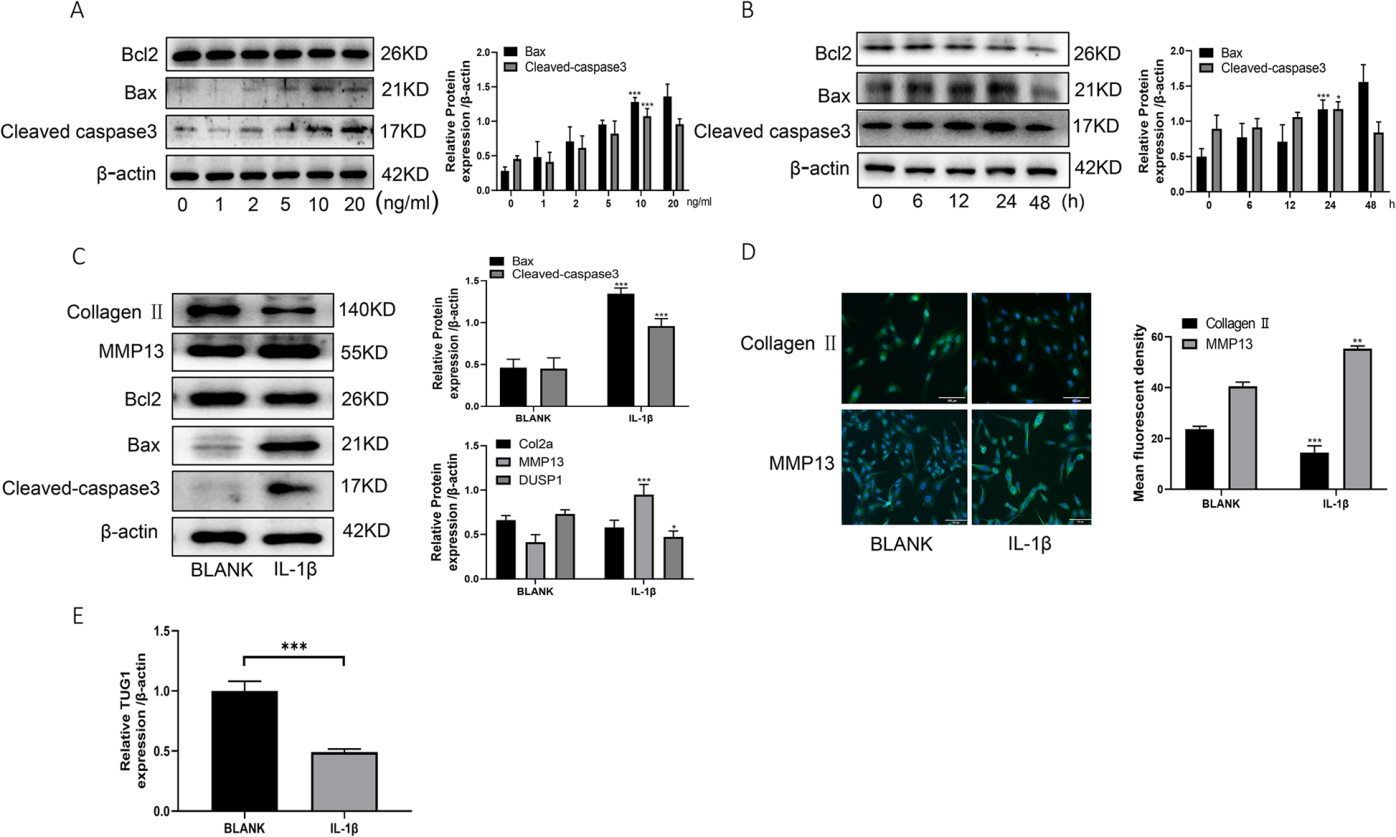
TUG1 is downregulated in IL-1β-induced C28/I2 chondrocyte cell line. **A** Western blotting to detect the protein expression of Bax, Bcl2, and cleaved-caspase3 at different concentrations of IL-1β stimulation. **B** Western blotting to detect the protein expression of Bax, Bcl2, and cleaved-caspase3 after different times of IL-1β stimulation. **C** Western blotting to detect the protein expression of Collagen II, MMP13, Bax, Bcl2, and cleaved-caspase3 after 10 ng/ml IL-1β stimulation for 24 h. **D** Immunofluorescence detection of collagen II and MMP13 protein expression after 10 ng/ml IL-1β stimulation for 24 h. **E** qRT-PCR assay of TUG1 mRNA expression after 10 ng/ml IL-1β stimulation for 24 h. All data are shown as SEM ± mean based on 3 independent experiments. *P < 0.05; **P < 0.01; *P* < 0.001

C28/I2 cells were treated with different concentrations (0, 1, 2, 5, 10, 20 ng/ml) of IL-1β for 24 h, followed by treatment of C28/I2 cells with 10 ng/ml IL-1β for different times (0, 6, 12, 24, 48 h) to detect apoptosis-associated protein expression (Fig. 2A, B). The optimal treatment concentration and time (10 ng/ml, 24 h) were selected to treat C28/I2 cells. Western blotting and immunofluorescence were used to detect the expression of apoptosis and cartilage damage-related proteins, and we found that IL-1β could induce apoptosis and extracellular matrix degradation in C28/I2 cells (Fig. 2C–E). qRT-PCR detected TUG1 expression, we found that TUG1 was also downregulated in IL-1β-treated C28/I2 cells (Fig. 2E). These results suggest that TUG1 may be involved in chondrocyte apoptosis and inflammation.

### Knockdown of TUG1 inhibits chondrocyte viability and promotes chondrocyte apoptosis and inflammation

**Fig. 3:**
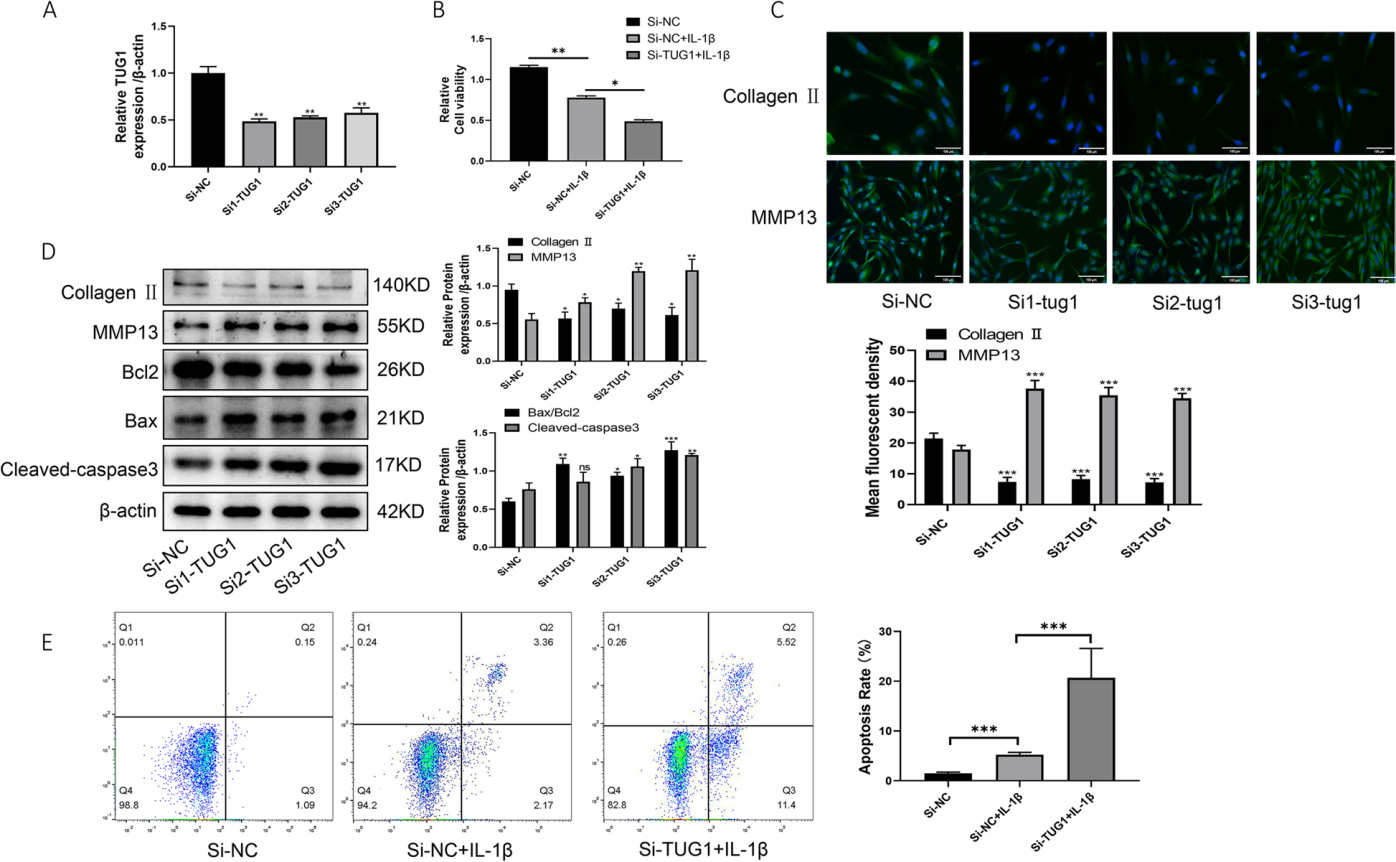
Knockdown of TUG1 inhibits chondrocyte viability and promotes chondrocyte apoptosis and inflammation. **A** qRT-PCR assay of TUG1 expression after interference using siRNA. **B** CCK-8 assay of Si-TUG1 for cellular value-added viability. **C** Immunofluorescence detection of collagen II and MMP13 protein expression of Si-TUG1. **D** Western blotting to detect the protein expression of collagen II, MMP13, Bax, Bcl2, and cleaved-caspase3 in Si-TUG1. **E** Flow cytometry detection of apoptosis in Si-TUG1. All data are shown as SEM ± mean based on 3 independent experiments. *P < 0.05; **P < 0.01; *P* < 0.001

To further investigate the effect of TUG1 on chondrocyte apoptosis and inflammation, we transfected C28/I2 cells with siRNA. qRT-PCR showed that the interference efficiency reached more than 50% (Fig. 3A). CCK-8 assay showed that interference with TUG1 inhibited the value-added of chondrocytes (Fig. 3B). Increased expression of Bax, cleaved-caspase3, and MMP13 proteins and decreased expression of Bcl2 and collagen II proteins after interfering with TUG1 (Fig. 3C, D). Annexin V-FITC/PI double staining combined with flow cytometry for detection of apoptosis (Fig. 3E). In conclusion, these findings suggest that the knockdown of TUG1 inhibits chondrocyte proliferation and promotes chondrocyte apoptosis and inflammation.

### miR144-3P is a target miRNA for TUG1

**Fig. 4:**
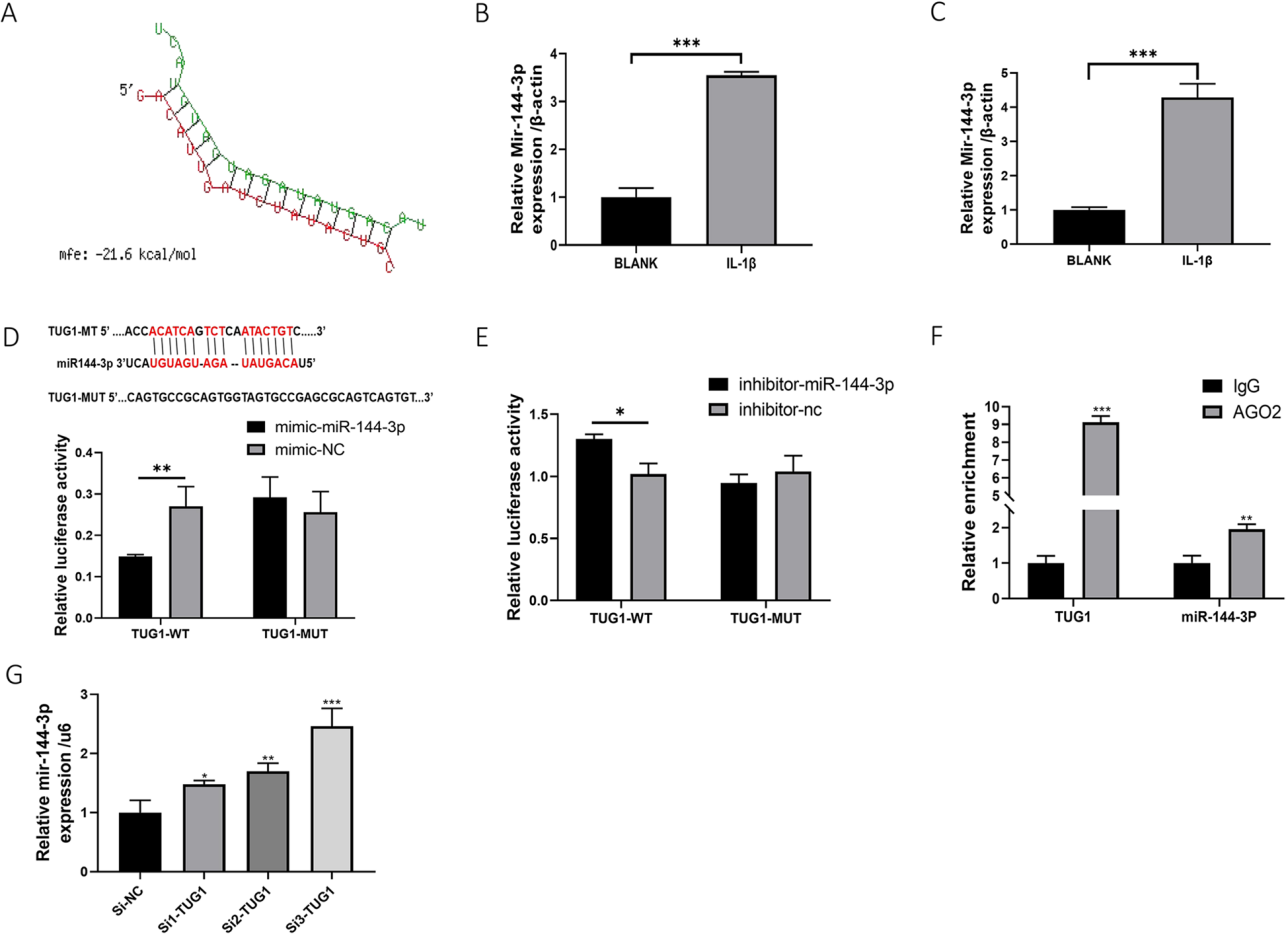
miR144-3P is a target miRNA for TUG1. **A** RNAhybrid database analysis reveals a potential interaction between TUG1 and miR144-3p. **B** qRT-PCR assay of miR144-3p expression in IL-1β-induced human primary chondrocytes. **C** qRT-PCR to detect IL-1β-induced miR144-3p expression in C28/I2 cells. **D**, **E** Targeted binding of TUG1 to miR144-3p was detected by double luciferase reporter gene after mimic and inhibitor cotransfection of TUG1-WT and TUG1-MUT with miR144-3p. **F** RIP assay for the binding of TUG1, AGO2 protein, and miR144-3p. **G** qRT-PCR assay for miR144-3p expression of Si-TUG1. All data are shown as SEM ± mean based on 3 independent experiments. *P < 0.05; **P < 0.01; *P* < 0.001

In our study, miR144-3P was found to be upregulated in both IL-1β-induced primary OA chondrocytes and C28/I2 cells (Fig. 4B, C). To test the relationship between miR144-3P and TUG1, the online database starBase2.0 was used to predict our hypothesis. miR144-3p was found to be a potential miRNA target of TUG1, and the potential interaction between TUG1 and miR144-3p was further revealed by RNAhybrid database analysis (Fig. 4A). As measured by the luciferase reporter gene, luciferase activity was significantly reduced when miR-144-3p bound to TUG1-wt, but not TUG1-mut (Fig. 4D). In contrast, luciferase activity was increased when the inhibitor against miR-144-3P was cotransfected with TUG1-wt (Fig. 4E). RIP experiments further validated that TUG1 directly targets binding to miR14-3P (Fig. 4F). Furthermore, interference of TUG1 significantly increased the expression of miR144-3P (Fig. 4G). Therefore, we conclude that miR144-3P is a target miRNA for TUG1.

### miR144-3P promotes apoptosis and inflammation in chondrocytes

**Fig. 5:**
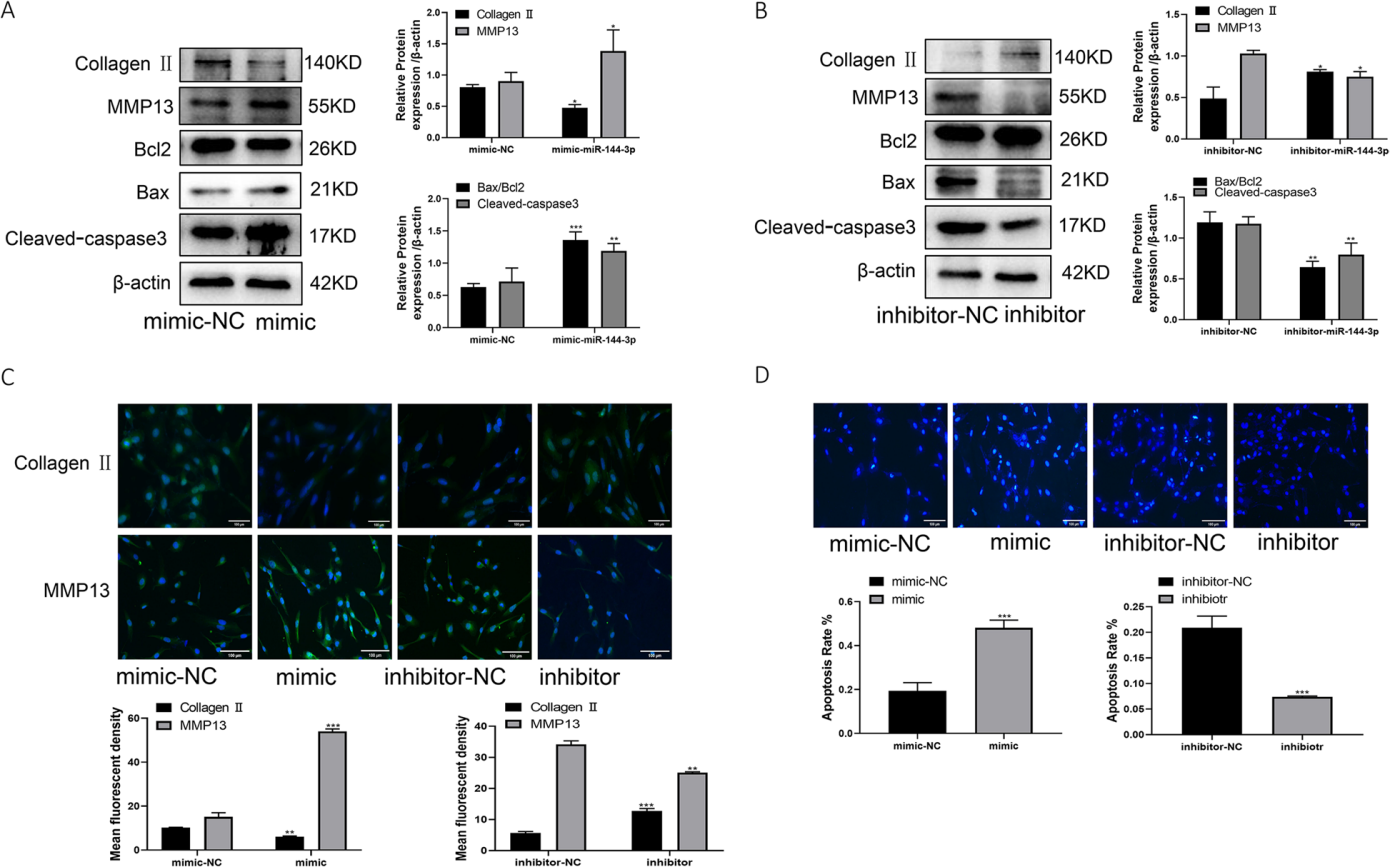
miR144-3P promotes apoptosis and extracellular matrix degradation in chondrocytes. **A** Western blotting to detect the protein expression of collagen II, MMP13, Bax, Bcl2, and cleaved-caspase3 after transfection with mimic. **B** Western blotting to detect the protein expression of collagen II, MMP13, Bax, Bcl2, and cleaved-caspase3 after transfection with inhibitor. **C** Immunofluorescence detection of collagen II and MMP13 protein expression after transfection with mimic, inhibitor. **D** Hochest33342 assay for apoptosis after transfection with mimic, inhibitor. All data are shown as SEM ± mean based on 3 independent experiments. *P < 0.05; **P < 0.01; *P* < 0.001

To investigate the role of miR144-3P in OA, we transfected mimic and inhibitor of miR144-3P into C28/I2 cells. Western blotting and immunofluorescence results showed that Bax, cleaved-caspase3, and MMP13 protein expression increased and Bcl2 and collagen II expression decreased in the mimic group. On the contrary, Bax, cleaved-caspase3, and MMP13 protein expression decreased and Bcl2 and collagen II protein expression increased in the inhibitor group (Fig. 5A–C). The results of Hochest33342 showed that apoptosis increased in the mimic group and decreased in the inhibitor group (Fig. 5D). Thus suggesting that upregulation of miR144-3P can promote apoptosis and inflammation in chondrocytes, while downregulation of miR144-3P can inhibit apoptosis and inflammation in chondrocytes.

### DUSP1 is a target mRNA for miR144-3P

**Fig. 6:**
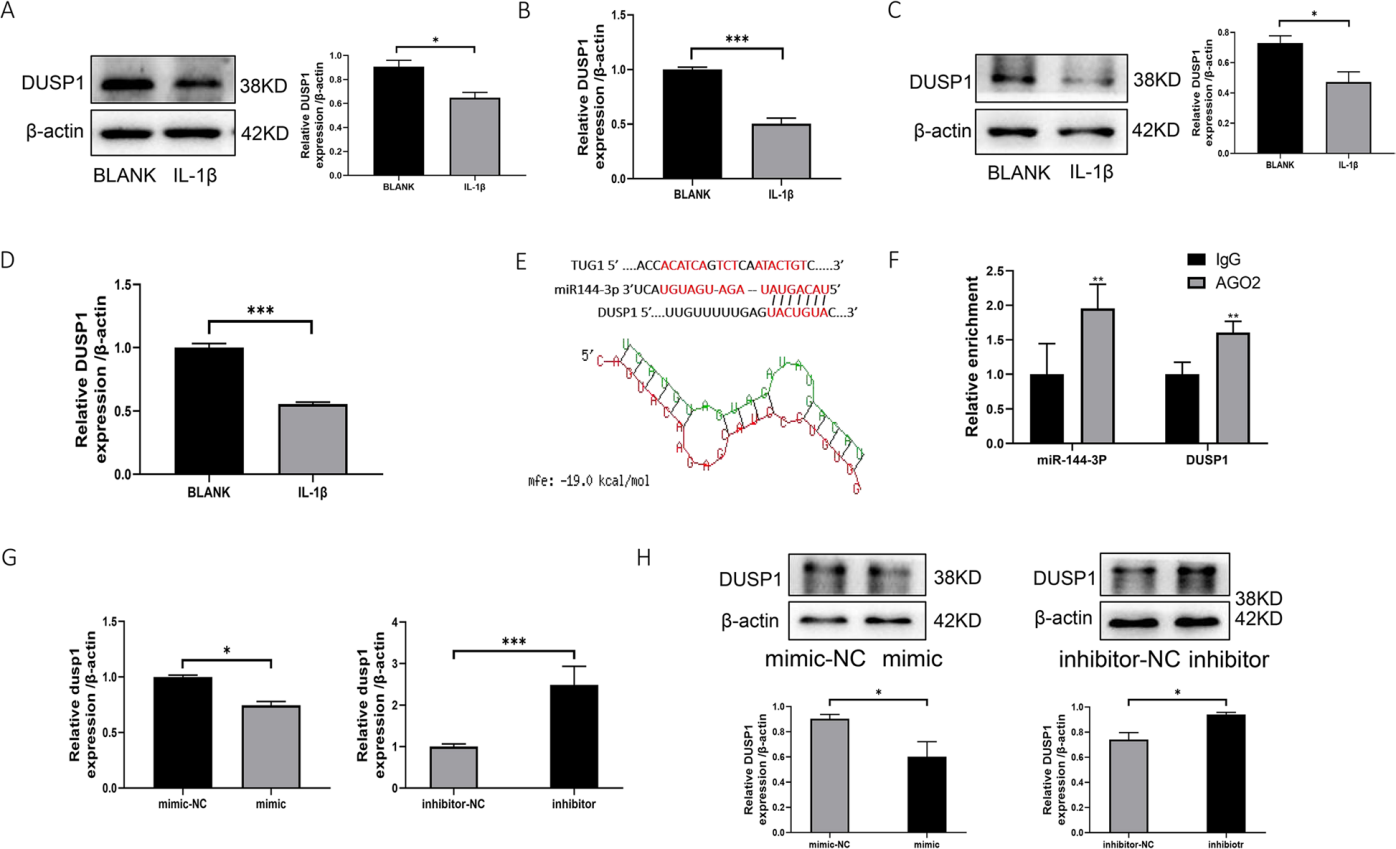
DUSP1 is the target mRNA of miR144-3P. **A** Western blotting to detect the protein expression of DUSP1 in IL-1β-induced human primary chondrocytes. **B** qRT-PCR to detect IL-1β-induced mRNA expression of DUSP1 in human primary chondrocytes. **C** Western blotting to detect the protein expression of DUSP1 in IL-1β-induced C28/I2 cells. **D** qRT-PCR to detect the mRNA expression of DUSP1 in IL-1β-induced C28/I2 cells. **E** RNAhybrid database analysis revealed a potential interaction between miR144-3P and DUSP1. **F** RIP assay for targeted binding of miR144-3P to DUSP1. **G** qRT-PCR to detect the mRNA expression level of DUSP1 after transfection with mimic, inhibitor. **H** Western blotting detection of protein expression levels of DUSP1 after transfection with mimic, inhibitor. All data are shown as SEM ± mean based on 3 independent experiments. *P < 0.05; **P < 0.01; *P* < 0.001

We found that DUSP1 was downregulated in both IL-1β-induced OA chondrocytes and C28/I2 cells (Fig. 6A–D). A binding site for miR-144-3p to the DUSP1 3′UTR was found by starBase2.0. The potential interaction between miR144-3p and DUSP1 was also revealed by RNAhybrid database analysis (Fig. 6E). RIP experiments further verified that DUSP1 directly binds miR144-3P (Fig. 6F). In addition, using qRT-PCR and Western blotting, mRNA and protein expression of DUSP1 was downregulated by overexpression of miR144-3P, while mRNA and protein expression of DUSP1 was upregulated by interference with miR144-3P (Fig. 6G, H). This indicates that DUSP1 is the target mRNA of miR144-3P.

### Overexpression of DUSP1 attenuates chondrocyte apoptosis and inflammation

**Fig. 7:**
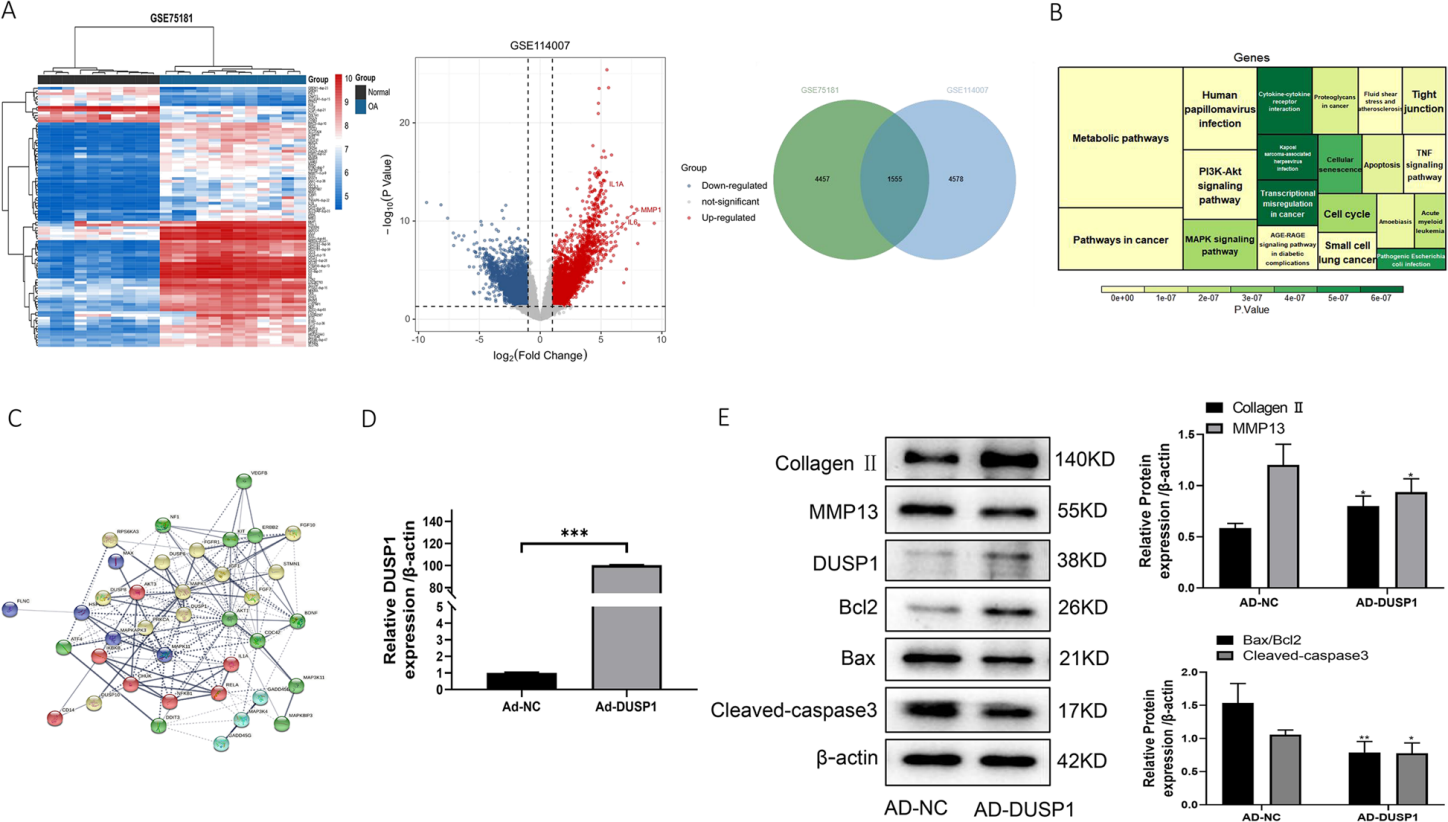
Overexpression of DUSP1 attenuates chondrocyte apoptosis and extracellular matrix degradation. **A** Differential analysis of datasets from the GEO database (GSE75181, GSE114007) using the limma package and taking intersections of differential genes. **B** KEGG enrichment analysis of the differential genes. **C** PPI analysis of the differential genes. **D** qRT-PCR to detect the mRNA expression level of DUSP1 after transfection with AD-DUSP1. **E** Western blotting to detect protein expression of DUSP1, collagen II, MMP13, Bax, Bcl2, and cleaved-caspase3 after transfection with AD-DUSP1. All data are shown as SEM ± mean based on 3 independent experiments. *P < 0.05; **P < 0.01; *P* < 0.001

It has been reported that DUSP1 inhibits the activation of the P38 MAPK pathway, thereby slowing the pathogenesis of osteoarthritis. We, therefore, performed differential analysis using datasets from the GEO database (GSE75181, GSE114007) and found that DUSP1 was significantly downregulated in osteoarthritic cartilage (Fig. 7A). We also performed KEGG enrichment analysis and PPI protein interaction network analysis, and we found that DUSP1 could be involved in osteoarthritis and showed a close association with MAPK pathway (Fig. 7B, C). Overexpression of DUSP1, detected by qRT-PCR and Western blotting, showed that overexpression of DUSP1 significantly reduced apoptosis and inflammation in chondrocytes compared to the control group (Fig. 7D, E).

### TUG1 regulates the course of osteoarthritis via the miR144-3P/DUSP1/p38 MAPK axis

**Fig. 8:**
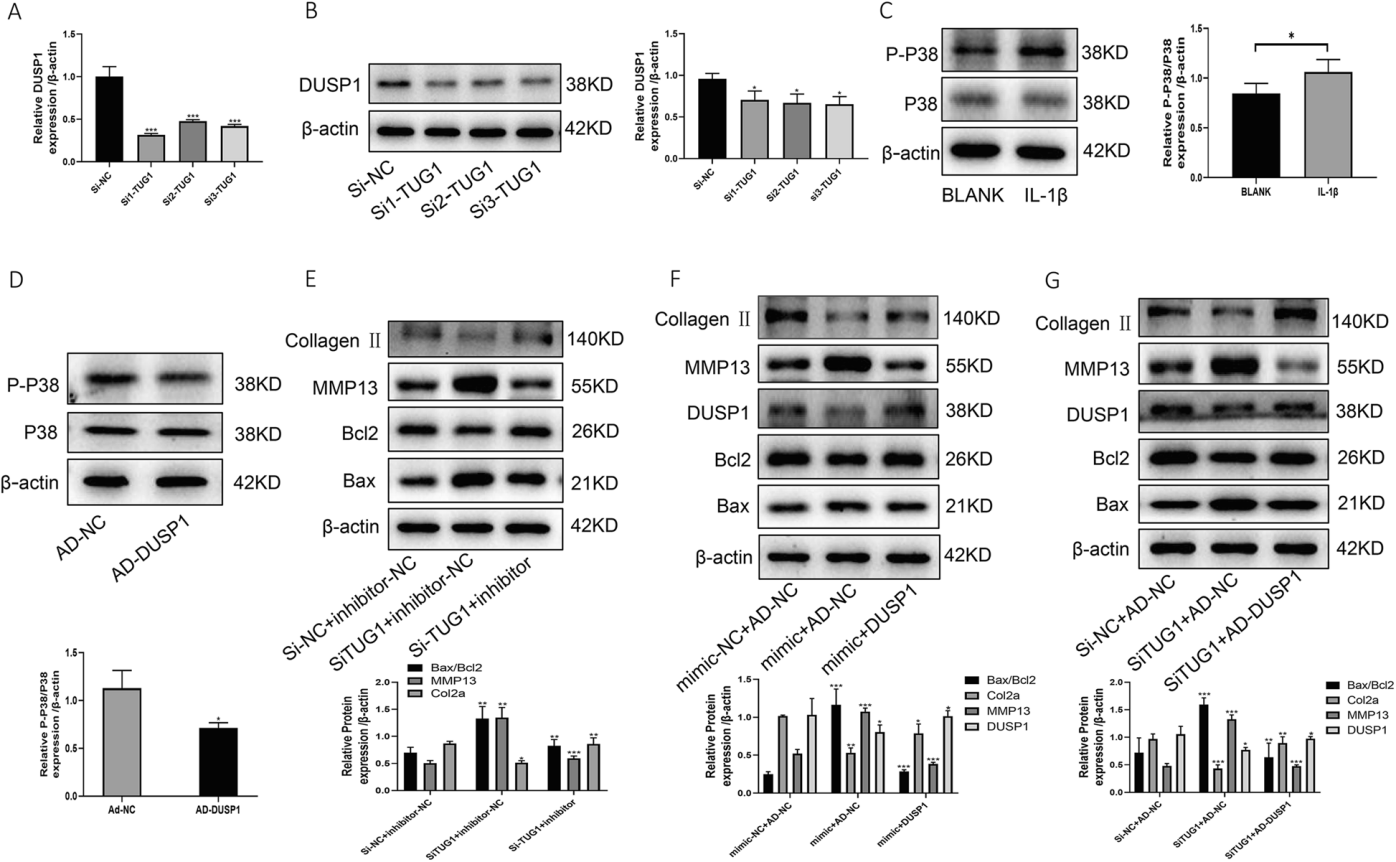
TUG1 regulates the course of osteoarthritis through the miR144-3P/DUSP1/p38 MAPK axis. **A** qRT-PCR to detect DUSP1 mRNA expression after transfection with Si-TUG1. **B** Western blotting to detect the protein expression of DUSP1 after transfection with Si-TUG1. **C** Western blotting to detect IL-1β-induced protein expression of P38 and P-P38 in chondrocytes. **D** Western blotting to detect P38 and P-P38 protein expression after transfection with AD-DUSP1. **E** Western blotting to detect the protein expression of collagen II, MMP13, Bax, and Bcl2 after cotransfection with Si-TUG1, miR144-3P inhibitor. **F** Western blotting to detect the protein expression of collagen II, MMP13, Bax, and Bcl2 after miR144-3P mimic, AD-DUSP1 cotransfection. **G** Western blotting to detect the protein expression of collagen II, MMP13, Bax, and Bcl2 after cotransfection with Si-TUG1, AD-DUSP1. All data are shown as SEM ± mean based on 3 independent experiments. *P < 0.05; **P < 0.01; *P* < 0.001

To verify that TUG1 regulates apoptosis and inflammation in OA chondrocytes by targeting miR-144-3p/DUSP1/P38 MAPK. Knockdown of TUG1, qRT-PCR with Western blotting results showed reduced protein expression of DUSP1 (Fig. 8A, B). Induction of chondrocytes using IL-1β activated the P38 MAPK pathway (Fig. 8C). In contrast, the overexpression of DUSP1 was able to inhibit the activation of the P38 MAPK pathway (Fig. 8D). By cotransfection with Ad-si-TUG1 + miR-144-3p inhibitor, miR-144-3p mimic + Ad-DUSP1, Ad-si-TUG1 + Ad-DUSP1, adding IL-1β induction. Western blotting detected Bax, Bcl-2, DUSP1, MMP13, and collagen II protein expression, the results showed that inhibition of miR144-3P overexpression reversed the chondrocyte apoptosis, and inflammation promoted by knockdown of TUG1, while overexpression of DUSP1 reversed the chondrocyte apoptosis and inflammation promoted by overexpression of miR144-3P, while overexpression of DUSP1 reversed the chondrocyte apoptosis and inflammation promoted by knockdown of TUG1 (Fig. 8E–G). We finally concluded that TUG1 was able to inhibit OA chondrocyte apoptosis and inflammatory response by competitively binding miR-144-3p, deregulating the negative regulatory effect of miR-144-3p on DUSP1, promoting DUSP1 expression and inhibiting the p38 MAPK signaling pathway.

## Discussion

Chondrocyte apoptosis and inflammatory response are important pathological mechanisms in the progression of osteoarthritis, and effective intervention in this process is key to the treatment of OA. In recent years, an increasing number of studies have shown that lncRNAs are involved in the degenerative process of OA chondrocyte damage. For example, ROCR contributes to SOX9 expression and cartilage differentiation in human mesenchymal stem cells [18]. MEG3 accelerates osteoarthritis progression by downregulating miR-16 [19]. Recent studies have found that TUG1 is involved in the regulation of many diseases. A novel network of regulatory mechanisms mediated by TUG1 induces remodeling injury in preeclamptic spiral arteries [20], and it is also aberrantly expressed in a variety of tumors, affecting tumor cell proliferation and apoptosis [21, 22]. Although studies on TUG1 regulation of cartilage damage and extracellular matrix degradation have been reported, most previous reports have focused on the regulation of cartilage phenotype genes by TUG1, while the link between TUG1 and signaling pathways associated with cartilage damage remains understudied. In our study, we examined the TUG1 expression in primary OA chondrocytes and C28/I2 cells by combining expression data from the GEO database and bioinformatics analysis and found that TUG1 is under-expressed in osteoarthritis and shows a close correlation with the P38 MAPK signaling pathway. Our study further identifies a role for TUG1 in regulating cartilage damage in osteoarthritis and correlates it with the MAPK pathway. This is consistent with previous reports that TUG1 can be involved in regulating apoptosis and proliferation through MAPK, NF-KB, and other pathways [23, 24]. This sets the stage for the subsequent exploration of other functions and modulations of TUG1 in osteoarthritic cartilage damage.

New studies have identified that miR-144-3p plays an important role in the development of tumor diseases. For example, miR-144-3p interferes with the progression of hepatocellular carcinoma by regulating EIF4G2 [25]. miR-144-3p also inhibits the progression of gastric cancer by directly targeting GLI2 [26]. In addition, miR-144-3p is also involved in regulating the proliferation of colorectal cancer cells [27], and one study found that miR-144-3p could improve osteoarthritis by targeting IL-1β, suggesting that miR-144-3p may have potential therapeutic implications for osteoarthritis [28]. Consistent with previous studies, we found that miR-144-3p was upregulated in both OA primary chondrocytes and C28/I2 cells and predicted that miR-144-3p could serve as a target gene for TUG1 by starbase, miRDB, and other databases. We verified that miR-144-3p could directly target and bind to TUG1 by double luciferase reporter gene and RIP. Moreover, knockdown of TUG1 increased the expression of miR-144-3p, while overexpression of miR-144-3p promoted chondrocyte apoptosis and inflammation, and conversely, interference with miR-144-3p inhibited chondrocyte apoptosis and inflammation. In addition to this, overexpression of miR-144-3p reversed the promotion of chondrocyte apoptosis and inflammation by the knockdown of TUG1. These results suggest that TUG1 can be involved in regulating chondrocyte apoptosis and inflammation by targeting miR-144-3p.

DUSP1 is an important negative regulator of the inflammatory response [29–31]. It has been shown that DUSP1 can control the activation and inactivation of the MAPK pathway by regulating p38 MAPK [32–34]. In our study, miR-144-3p was found to regulate apoptosis and inflammation in OA chondrocytes by targeting DUSP1. We predicted the existence of binding sites for miR-144-3p to DUSP1 through Starbase, miRDB, and other databases. We verified by RIP that miR-144-3p and DUSP1 could directly target binding. In addition, overexpression of miR-144-3p decreased DUSP1 expression and, conversely, knockdown of miR-144-3p increased DUSP1 expression. Overexpression of DUSP1 inhibited chondrocyte apoptosis and inflammation and reversed the promotion of chondrocyte apoptosis and inflammation by miR-144-3p overexpression and by TUG1 knockdown. Interference with TUG1 inhibited DUSP1 expression and increased miR-144-3p expression. Consistent with previous studies, IL-1β induced activation of the P38 MAPK pathway, whereas overexpression of DUSP1 inhibited activation of the P38 MAPK pathway. These results strongly suggest that TUG1 may be involved in regulating the progression of OA by targeting miR-144-3P to regulate DUSP1/P38 MAPK.

However, our study still has several limitations. Firstly, we only explored the molecular mechanism of TUG1 as ceRNA sponging miR-144-3p; however, whether TUG1 can act as ceRNA to affect the expression of other key regulators in osteoarthritis needs to be further investigated. Secondly, besides playing the function of ceRNA, whether TUG1 is involved in regulating the progression of osteoarthritis through other functions. Thirdly, limited by gene germline issues, we have only investigated the function of TUG1 in vitro. Next, we will further explore the functions of TUG1 in osteoarthritic cartilage damage and extracellular matrix degradation, with a focus on TUG1 and cellular signaling pathways.

## Conclusions

In summary, this study clarified the specific molecular biological link of TUG1 on apoptosis and inflammatory response in OA chondrocytes and validated the ceRNA regulatory network of TUG1/miR-144-3p/DUSP1/P38 MAPK in chondrocytes. In conclusion, our study provides an experimental and theoretical basis for promoting arthritic cartilage repair employing genetic engineering.

## Supplementary Information

**Additional file 1.**

## Data Availability

The datasets generated during and/or analyzed during the current study are available in the Gene Expression Omnibus repository [https://www.ncbi.nlm.nih.gov/geo/].

## Abbreviations

OA: Osteoarthritis
lncRNA: Long non-coding RNA
TUG1: Taurine upregulated 1
DUSP1: Dual specificity phosphatase 1
P38 MAPK: P38 mitogen-activated protein kinase
miRNA: MicroRNA
RIP: RNA-binding protein immunoprecipitation assay
SiRNA: Small interfering RNA
si-TUG1: Interfering RNA of TUG1
Ad-DUSP1: Overexpression plasmid of DUSP1
AGO2: Argonaute2
IgG: Immunoglobulin G
PBS: Phosphate-buffered saline
BSA: Bovine serum albumin
GEO: Gene Expression Omnibus
MMP13: Matrix metallopeptidase 13
Bax: BCL2-associated X
Bcl2: B-cell lymphoma-2
NC: Negative control
SEM: Standard error
TUG1-wt: Wild-type TUG1 overexpression plasmid
TUG1-mut: Mutant TUG1 overexpression plasmid
